# Structural Insight into Inhibitor of Apoptosis Proteins Recognition by a Potent Divalent Smac-Mimetic

**DOI:** 10.1371/journal.pone.0049527

**Published:** 2012-11-15

**Authors:** Federica Cossu, Mario Milani, Patrice Vachette, Francesca Malvezzi, Serena Grassi, Daniele Lecis, Domenico Delia, Carmelo Drago, Pierfausto Seneci, Martino Bolognesi, Eloise Mastrangelo

**Affiliations:** 1 Department of Biosciences, University of Milano, Milano, Italy; 2 CNR-Istituto di Biofisica, Università degli Studi di Milano, Milano, Italy; 3 Institut de Biochimie et de Biophysique Moléculaire et Cellulaire, UMR8619 CNRS, Université Paris-Sud, IFR115, Orsay, France; 4 Istituto Nazionale dei Tumori, Milano, Italy; 5 Centro Interdisciplinare Studi bio-molecolari e applicazioni Industriali (CISI), University of Milano, Milano, Italy; 6 Department of Organic and Industrial Chemistry, University of Milano, Milano, Italy; IISER-TVM, India

## Abstract

Genetic alterations enhancing cell survival and suppressing apoptosis are hallmarks of cancer that significantly reduce the efficacy of chemotherapy or radiotherapy. The Inhibitor of Apoptosis Protein (IAP) family hosts conserved proteins in the apoptotic pathway whose over-expression, frequently found in tumours, potentiates survival and resistance to anticancer agents. In humans, IAPs comprise eight members hosting one or more structural Baculoviral IAP Repeat (BIR) domains. Cellular IAPs (cIAP1 and 2) indirectly inhibit caspase-8 activation, and regulate both the canonical and the non-canonical NF-κB signaling pathways. In contrast to cIAPs, XIAP (X chromosome-linked Inhibitor of Apoptosis Protein) inhibits directly the effector caspases-3 and -7 through its BIR2 domain, and initiator caspase-9 through its BIR3 domain; molecular docking studies suggested that Smac/DIABLO antagonizes XIAP by simultaneously targeting both BIR2 and BIR3 domains. Here we report analytical gel filtration, crystallographic and SAXS experiments on cIAP1-BIR3, XIAP-BIR3 and XIAP-BIR2BIR3 domains, alone and in the presence of compound 9a, a divalent homodimeric Smac mimetic. 9a is shown to bind two BIR domains inter- (in the case of two BIR3) and intra-molecularly (in the case of XIAP-BIR2BIR3), with higher affinity for cIAP1-BIR3, relative to XIAP-BIR3. Despite the different crystal lattice packing, 9a maintains a right handed helical conformation in both cIAP1-BIR3 and XIAP-BIR3 crystals, that is likely conserved in solution as shown by SAXS data. Our structural results demonstrate that the 9a linker length, its conformational degrees of freedom and its hydrophobicity, warrant an overall compact structure with optimal solvent exposure of its two active moieties for IAPs binding. Our results show that 9a is a good candidate for pre-clinical and clinical studies, worth of further investigations in the field of cancer therapy.

## Introduction

Apoptosis is a process of programmed cell death essential for homeostasis maintenance in multicellular organisms, which is regulated by a subset of caspases (Cysteine-dependent ASPartyl-specific proteASES) in charge of propagating, once activated, the apoptotic signal to the nucleus [1]. The suppression of caspase activity occurs in the presence of specific members of the IAP (Inhibitor of Apoptosis Proteins) family [2], [3]. In particular, cIAP1 and cIAP2 (cellular IAPs) are indirect inhibitors of caspases activity, whereas XIAP (X chromosome-linked Inhibitor of Apoptosis Protein) is able to directly inhibit both initiator and effector caspases. All IAPs host one to three BIR (Baculoviral IAP Repeat) domains that are critical for their anti-apoptotic activity. In particular, it has been shown that the XIAP-BIR2 domain is responsible for the inhibition of effector caspases, whereas XIAP-BIR3 directly binds to and inhibits initiator caspase-9, which can also be recognized by cIAP1-BIR3 [4]. The caspase inhibitory activity of XIAP is endogenously antagonized by Smac/DIABLO (Second mitochondria-derived activator of caspases/Direct IAp Binding protein with Low pI), which is released from mitochondria together with cytochrome *c* in response to death stimuli. The N-terminal tetrapeptides of Smac/DIABLO and caspases (known as Iap Binding Motives, IBMs) competitively bind to the same XIAP active pocket (the IBM binding cleft), resulting in activation or inhibition of apoptosis, respectively. Since the structural details of IBM interactions with XIAP and cIAPs have been previously described [4], [5], the IBM peptides provide a natural basis for the design of Smac-mimetics. These compounds have been shown to displace caspases 3, 7 and 9 from XIAP-BIR2 and –BIR3 inhibitory pockets, and to induce auto-ubiquitination and degradation of cIAPs by perturbing BIR3/RING domain interaction [6], [7]. Therefore, the Smac-mimetics can restore the apoptotic cascade operating in a variety of signaling pathways.

Over the last few years several Smac-mimetics have been designed (based on the Smac/DIABLO N-terminal tetrapeptide AVPI), with the aim of exploiting their pro-apoptotic properties, alone or in combination with other pro-apoptotic compounds such as TRAIL [8]; these initiatives led to the progressive development of new and potent compounds, some of which are currently in phase I clinical trials [9]. One of the most promising Smac-mimetics is SM164, a divalent molecule composed of two moieties, connected by a flexible linker, aimed to target simultaneously two BIR domains [10].

**Figure 1:**
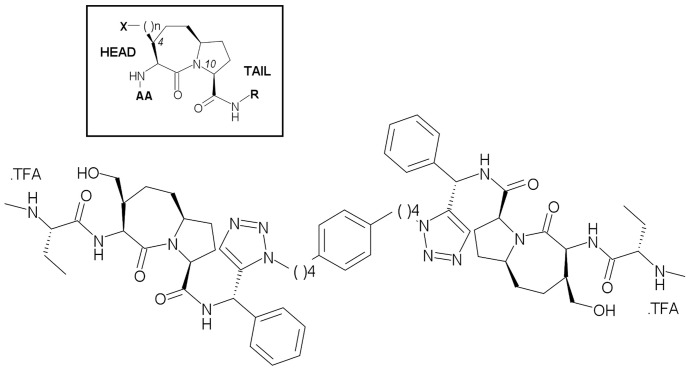
Chemical structure of tail-tail dimer 9a. The left inset shows a framed structure of the divalent Smac-mimetics based on the 1-aza-2-oxobicyclo[5.3.0]decane scaffold.

Taking advantage of the experience gathered with monovalent Smac-mimetics design [11], [12], we generated a library of twenty divalent compounds, belonging to three structural sub-classes, each characterized by distinct linkers or central scaffold-substitutions, to explore different molecular rigidity patterns and to test related metabolic assumptions [13], [14]. All divalent compounds were fully profiled *in vitro*, and compared in terms of overall drug-like properties. In particular, 9a (Fig. 1) displayed *in vitro* low nM affinity values for the BIR3 domains of XIAP, cIAP1 and cIAP2, but also for XIAP-BIR2BIR3; it also showed good cytotoxicity properties against a selected breast cancer cell line. Notably, due to its ionisable secondary amino groups, 9a is soluble in physiological buffer and could be administered *in vivo*; thus, it resulted as the most promising compound in our library, and was selected for early *in vivo* characterization [13], [14]. 9a displayed significant potency as a single agent in reducing the development of solid tumours in mice injected subcutaneously with a human ovarian cancer cell line, and increased the median survival time of mice in a human ovarian ascites model [14].

In this communication we present biochemical, biophysical and structural characterization of 9a in its complexes with XIAP-BIR3, XIAP-BIR2BIR3 and cIAP1-BIR3. In particular, we report data on compound 9a binding to different BIR domains through analytical gel filtration and small angle X-ray scattering (SAXS). Moreover, we present the crystal structures of cIAP1-BIR3 and XIAP-BIR3 domains in the presence of 9a, describing the molecular details of divalent Smac-mimetic recognition. Taken together, all the experimental evidences here reported suggest that 9a is one of the most powerful divalent Smac-mimetics known to date; the structural analysis of its recognition patterns, here presented, is the basis for further optimization in terms of target affinity and bioavailability.

## Results

### Cellular Cytotoxicity

Preliminary cytotoxicity tests of 9a after 72 hours of treatment *versus* MDA-MB-231 (a breast cancer cell line that has repeatedly been used to test Smac-mimetics/XIAP inhibitors), HL60 (known to be Smac-mimetic sensitive), and PC-3 cells (as an example of Smac-mimetic refractory cells), were addressed [14]. 9a showed nanomolar cytotoxicity both in MDA-MB-231 and HL60 cell lines, whereas it was inactive against the PC-3 cell line, as expected. The relative IC_50_ values observed are shown in Table 1.

**Table 1:** Cytotoxic activity displayed by 9a on MDA-MB-231, HL60 and PC-3 cell lines, determined in three independent experiments (each done in triplicate).

Cytotoxicity IC_50_ [nM]			Fluorescence Binding Assays IC_50_ [nM]			
MDA-MB-231	HL60	PC-3	XIAP-BIR3	XIAP-BIR2BIR3	cIAP1-BIR3	cIAP2-BIR3
55±1.0	71±5.0	>50.000	25.4±0.1	0.8±0.1	5.4±0.1	1.9±0.9

*In vitro* IC_50_ values for 9a in complex with XIAP-BIR3, XIAP-BIR2BIR3, cIAP1-BIR3, cIAP2-BIR3 determined by 3 independent experiments.

### Caspase Activation and cIAP Degradation

**Figure 2:**
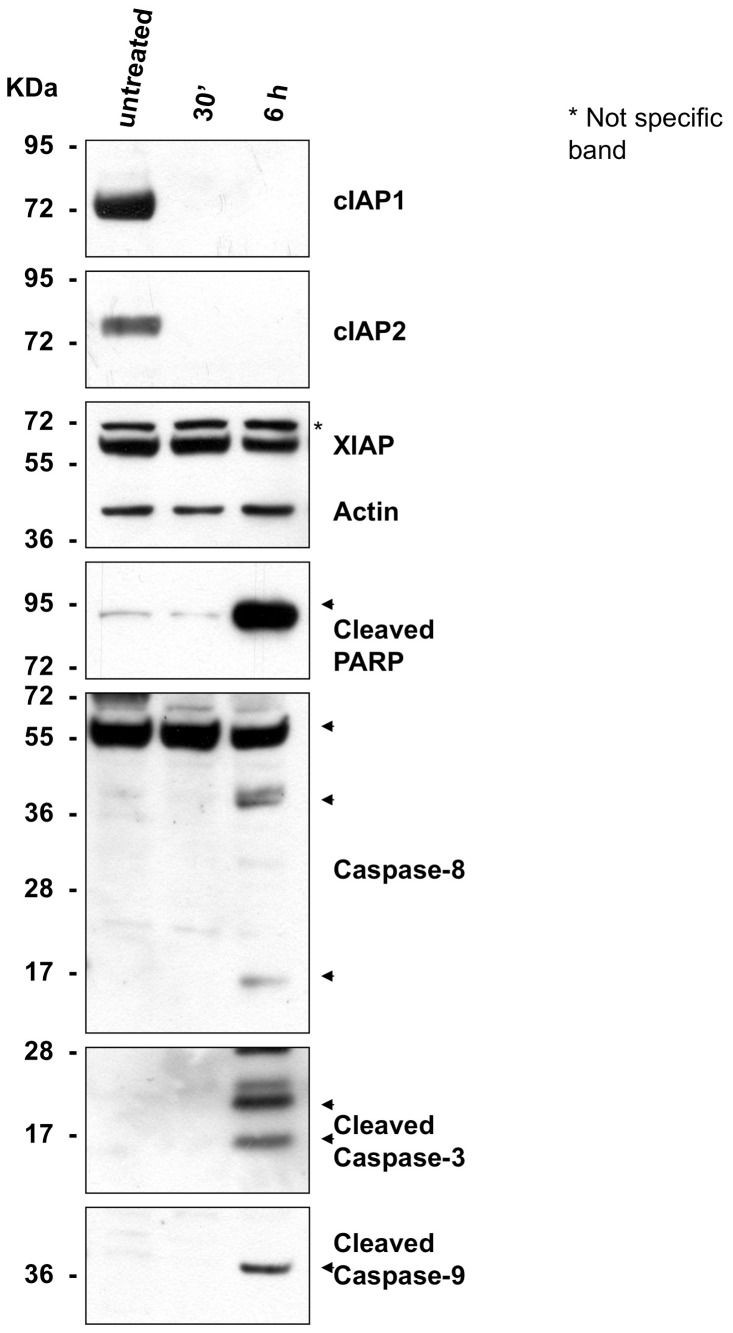
Western blot of MDA-MB-231 cells untreated or treated with 9a. Upper part: cIAP1 and cIAP2 degradation in the MDA-MB-231 cell line after 30 min and 6h of treatment with 9a. Proteins were revealed by Western blot using polyclonal antibodies specific for cIAP1 and cIAP2. Lower part: activation of caspase-8 (arrows indicate p55, p43/41 and p18 forms), -9 (p37) and -3 (p17 and p19) by 9a (p89). Proteins were revealed by Western blot using rabbit polyclonal antibodies specific for cleaved Parp, and cleaved caspase-8, -9 and -3. Prestained Protein SHARPMASS V (11−250 kDa; EuroClone) was used as molecular weight marker.

To test the capability of inducing caspase activation and apoptosis, MDA-MB-231 cells were treated with 9a, or left untreated. 9a not only inhibited cell growth in the MDA-MB-231 cell line, but Western blot analysis showed activation of caspase-8, -3 and -9, and apoptosis (Fig. 2). Moreover, Western blot analysis shows that 9a induces degradation of cIAP1 and of cIAP2 (Fig. 2), already at 30 min post treatment.

### Fluorescence Polarization Assays

Binding and displacement assays based on fluorescent polarization were used to evaluate the affinities of 9a for human cIAP1-, cIAP2-, XIAP-BIR3 and XIAP-BIR2BIR3 domains. Saturation binding experiments were performed to determine the binding affinity of the fluorescent probes to the IAP constructs of interest, as previously reported [11], [15], [16]. Competitive binding assays revealed that 9a displayed low nanomolar IC_50_ values for all tested IAP constructs (Table 1).

### Analytical Gel Filtration

**Figure 3:**
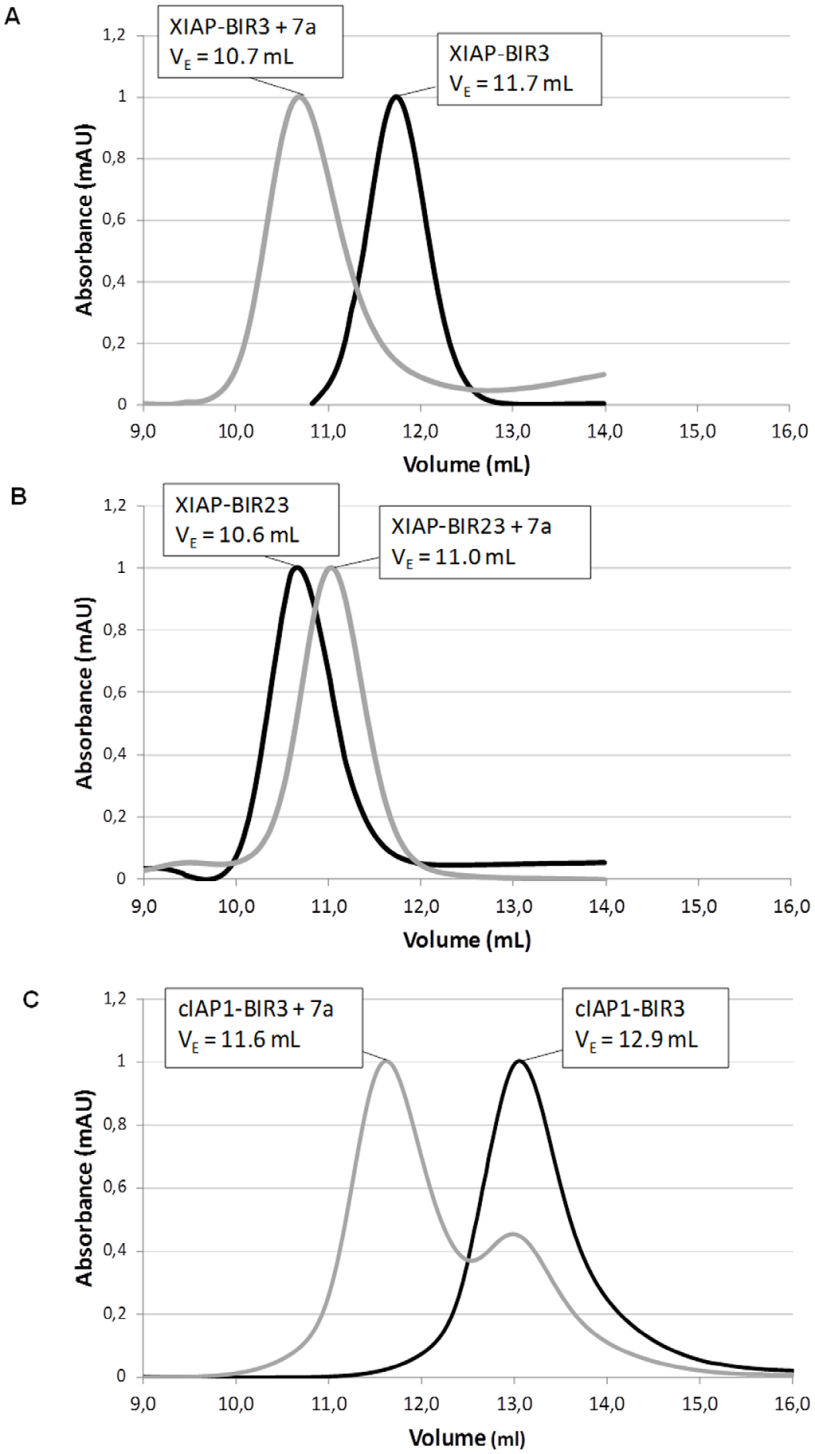
Analytical Gel Filtration Chromatograms. A) XIAP-BIR3 (33 µM) in absence/presence of an excess (1 mM) of 9a. B) XIAP-BIR2BIR3 in absence/presence of an excess (1 mM) of 9a. C) cIAP-BIR3 in absence/presence of an equal amount (33 µM) of 9a.F

In order to check whether simultaneous interactions of the divalent inhibitor with two cIAP1-BIR3 or XIAP-BIR3 domains could take place in solution, we performed analytical gel filtration assays mixing the different protein domains (33 µM) with a large excess of 9a (1 mM). The chromatograms obtained for XIAP-BIR3 in the presence of 9a exhibited a shift of 1 mL in elution volume (Ve = 10.7 mL) relative to the untreated protein (Ve = 11.7 mL), revealing domain dimerization upon ligand binding (Fig. 3A). In order to exclude that the dimerization of XIAP-BIR3 could be due to the formation of an intermolecular disulfide bridge involving residue Cys351 (induced by the presence of 9a), we performed an analytical gel filtration on the XIAP-BIR3 Cys351Ser mutant, in the absence/presence of the divalent compound, obtaining the same results reported for the wild type protein (Ve = 11.7/10.7 mL, respectively). In contrast, the chromatogram of XIAP-BIR2BIR3 exhibited a slight peak shift (0.3 mL) toward a higher elution volume in the presence of 9a, indicating a more compact conformation of the protein (Fig. 3B). The simultaneous binding of the two heads of divalent 9a to BIR2 and BIR3 domains, resulting in a decrease of their mutual distance, may explain the observed Ve shift (see also the SAXS data below). As expected, analytical gel filtration performed in the presence of a monovalent moiety of 9a did not show any peak shift relative to the apo-proteins.

Surprisingly, analytical gel filtration assays performed on cIAP1-BIR3 did not show any peak shift in the presence of an excess of 9a. To shed light on such behavior, we performed new gel filtration experiments mixing equimolar amounts (33 µM) of the proteins (XIAP- or cIAP1-BIR3) and of 9a. The resulting chromatograms showed for XIAP-BIR3/9a the same peak shift observed in the presence of an excess of inhibitor, and a peak shift of about 1.3 mL for cIAP1-BIR3/9a (from Ve = 12.9 mL to Ve = 11.6 mL, Fig. 3C).

Such apparently contradictory results can be explained taking into account the different affinities of the cIAP1- and XIAP-BIR3 domains for 9a in two different states: 1) when the ligand is free in solution; 2) when the ligand is already bound to one BIR3 domain, as summarized by the dissociation constants *K_1_* and *K_2_*:


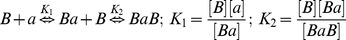
 *B* = BIR3, *a* = 9a

In fact, after mixing together the protein and a large excess of the inhibitor, an expected behavior (*K_1_*∼*K_2_*) will be the saturation of all the available BIR3 domains by one head of 9a, hampering the formation of dimers (no variations in Ve), as observed for cIAP1-BIR3. On the contrary if BIR3 has a considerably higher affinity for the BIR3/9a complex (*K_1_*>>*K_2_*), the equilibrium would be shifted toward dimer (*BaB*) formation, as observed for XIAP-BIR3 (variation in Ve). On the other hand, when the amount of inhibitor is comparable with that of the protein, there will be an equilibrium between the monomeric and dimeric adducts even if *K_1_*∼*K_2_* (Fig. 3C). As a whole, the analytical gel filtration results indicate that 9a is able to bind simultaneously two BIR domains (either BIR2 or BIR3), and bring them to a relatively compact (dimeric for XIAP-BIR3 and cIAP1-BIR3) structure.

### Crystal Structures of XIAP- and cIAP1-BIR3 Bound to 9a

#### Overall structure

The binding of 9a to the BIR3 domains of XIAP and cIAPs was investigated through X-ray crystallography. The 3D structures of cIAP1- and XIAP-BIR3 complexes with the ligand were solved through the molecular replacement method, using search models based on the BIR3 structure of cIAP1 (pdb: 3MUP [15]) and of XIAP (pdb: 3CLX [11]) and refined at 2.6 Å and 3.3 Å resolution, respectively (Table 2).

**Table 2:** X-ray data-collection and refinement statistics for the cIAP-BIR3/9a and XIAP-BIR3/9a complexes.

	cIAP-BIR3/9a	XIAP-BIR3/9a
Space group	C2	P2_1_2_1_2_1_
Unit-cell parameters (Å)	a = 79.1, b = 81.3, c = 96.9; β = 95.7°	a = 65.4, b = 130.5, c = 215.7
N° of molecules per a.u.	4	10
Resolution (Å)	56.6–2.6	54.0–3.3
Mosaicity (°)	0.8	0.5
N° of unique reflections	18,610 (2,731)	28,624 (4,112)
Completeness (%)	98.5 (99.5)	99.9 (100.0)
Redundancy	2.7 (2.7)	3.5 (3.6)
Rmerge † (%)	13.4 (58.5)	25.8 (50.8)
Average *I*/σ (*I*)	5.1 (1.6)	5.0 (2.6)
R factor ‡ (%)	25.7	21.0
Rfree § (%)	32.2	27.6
r.m.s. bond lengths (Å)	0.009	0.009
r.m.s. bond angles (°)	1.19	1.28
Average protein *B* factor (Å^2^)	52.8	56.3
Average Smac-mimetic *B* factor (Å^2^)	57.4	46.0
Residues in most favoured regions (%)	87,9%	85,2%
Residues in additionally allowed regions (%)	10,9%	14,8%
PDB-ID	4EB9	4EC4

Values in parentheses are for the highest resolution shell.

† **R_merge_** = Σ |*I* - (*I*)|/Σ*I* × 100, where *I* is intensity of a reflection and (*I*) is its average intensity.

‡ **R _factor_** = Σ |F_o_ - F_c_|/Σ |F_o_| × 100.

§ **R_free_**is calculated on 5% randomly selected reflections, for cross-validation.

**Figure 4:**
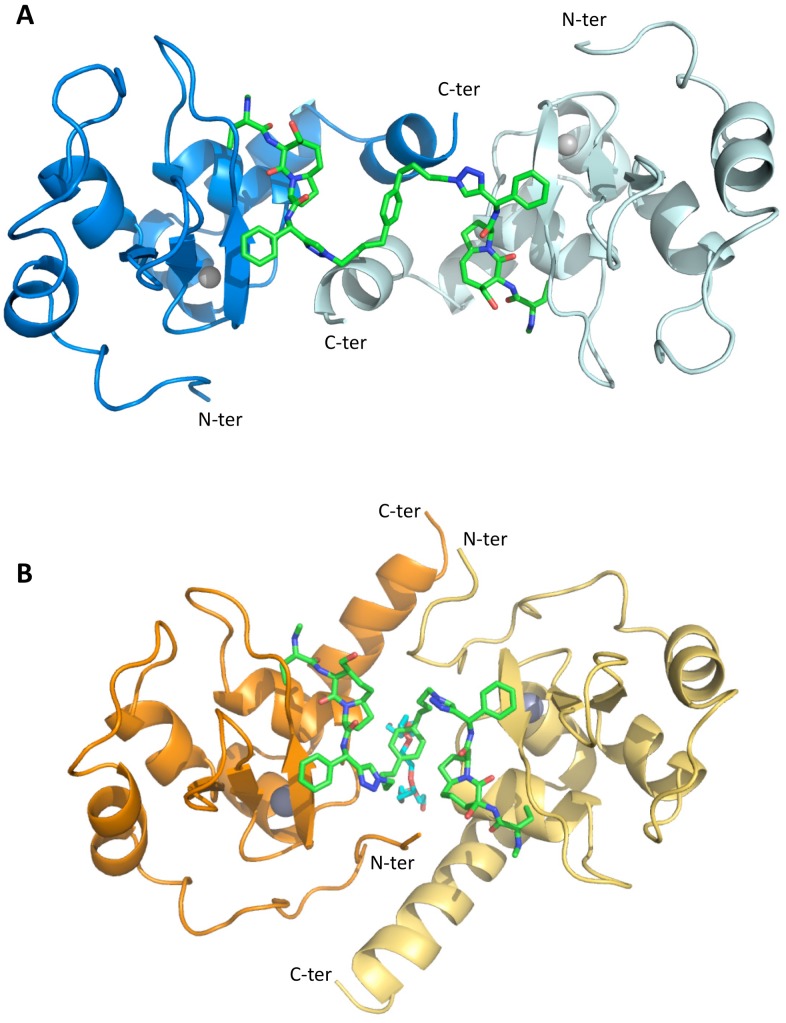
Dimeric assemblies of cIAP1- and XIAP1-BIR3 bound to 9a. A) X-Ray structure of cIAP1-BIR3 dimer (cartoon in blue and pale blue) in complex with 9a (green sticks). B) X-Ray structure of XIAP-BIR3 dimer in complex with 9a: the A and F molecules are in orange and pale yellow, respectively, 9a is represented as green sticks (drawn with Pymol).

In the cIAP1-BIR3 structure, the crystal displays four BIR3 domains and two molecules of 9a in the asymmetric unit, forming a ring-like assembly composed of two dimers (AC and BD; Fig. 4A shows the AC dimer). The crystal packing is the same observed for the structure of cIAP1-BIR3 bound to a monovalent Smac-mimetic (pdb: 3MUP; [15]), suggesting that such intermolecular arrangement is independent of the presence of the divalent compound. The four independent BIR3 domains display very similar structures, showing r.m.s.d. values in the 0.29 – 0.46 Å range (calculated over 101 Cα pairs).

In the XIAP-BIR3 structure, ten molecules in the asymmetric unit are assembled into five dimers (AF, BG, CJ, DK, EL), each arranged around a local twofold axis, in head-to-tail fashion, stabilized by the bound divalent 9a (Fig. 4B, AF dimer).

**Figure 5:**
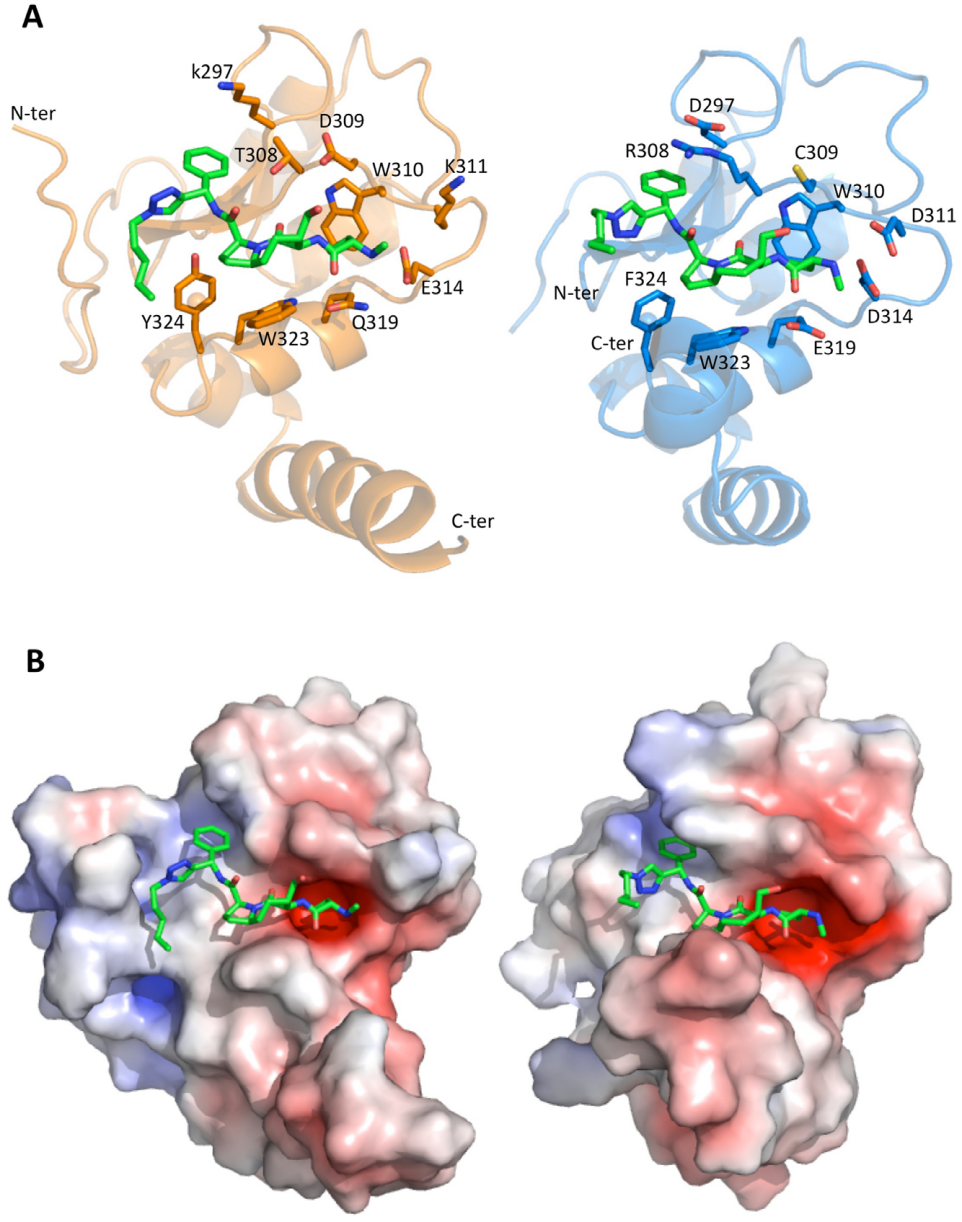
cIAP1- and XIAP1-BIR3 bound to one head of 9a. A) Left panel, XIAP-BIR3 (orange cartoon) with 9a (green sticks). The main residues involved in ligand interaction are shown in orange sticks; right-panel, cIAP-BIR3 (blue cartoon) with 9a (green sticks) and the main residues involved in ligand interaction (blue sticks). B) Left and right panels as A) with protein surface coloured by electrostatic potential (calculated using APBS2; drawn with Pymol).

The divalent Smac-mimetic heads bind to both cIAP1-BIR3 and XIAP-BIR3 in the conserved IBM cleft, between the β3 strand and the α3 helix, roughly lined by residues Gly306, Arg/Thr308, Cys/Asp309, Glu/Lys311, Asp/Glu314, Glu/Gln319 and Trp323, in cIAP1/XIAP-BIR3, respectively (Fig. 5A, left and right panels). In both structures the two heads of the ligand adopt antiparallel orientations, with distances of 17.0 and 11.8 Å between the N1 atoms of their triazole rings, for cIAP1- and XIAP-BIR3, respectively (Fig. 4A and B). In both cases the overall structure adopted by 9a is a sort of right-handed helix with different pitches due mainly to the rotation of ∼180° of the respective triazole rings (Fig. 4A and B).

#### The inhibitor linker region

The segment linking the two inhibitory heads of 9a (starting from the triazole ring) provides few hydrophobic contacts to the protein that do not seem to influence the recognition of the BIR3 IBM pocket by the Smac-mimetic. In the case of XIAP-BIR3, the 9a central phenyl ring, orthogonal to the dimer twofold axis (Fig. 4B), is hosted in a cleft between two BIR3 molecules surrounded by the N-terminal residues Asn249 and Pro251, and by the aromatic residues Trp323 and Tyr324. The interaction of 9a with XIAP-BIR3 N-terminal region promotes order in the N-terminal amino acids (248–253) that could be modelled in the electron density. In cIAP1 the linker segment is in contact with a hydrophobic surface built by the C-terminal α-helices of two BIR3 domains, in particular by residues Leu354 and Leu355.

### SAXS Analysis of XIAP-BIR2BIR3 with/without 9a

**Figure 6:**
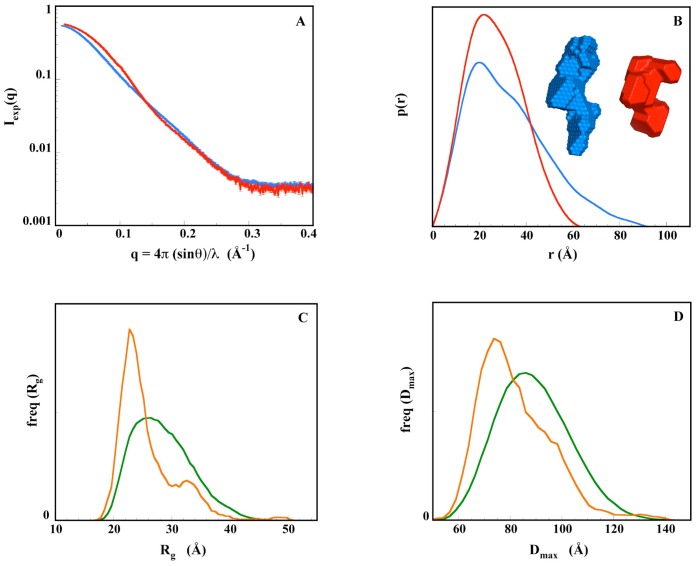
SAXS study of XIAP-BIR2BIR3. A) experimental scattering patterns with associated error bars; blue line: free XIAP-BIR2BIR3; red line: XIAP-BIR2BIR3 complexed with 9a. B) distance distribution functions p(r); color code as in panel A. C) distribution of Rg values of free XIAP-BIR2BIR3; green: random pool; orange: selected ensembles fitting the data; D) distribution of D_max_ values; color code as in panel C.

The two scattering patterns of XIAP-BIR2BIR3 in solution, in the absence/presence of 9a, are shown in Fig. 6A. They result from the combination of data recorded using an on-line HPLC apparatus to ensure protein monodispersity (small-angle data), with data from a higher concentration sample (wide-angle), as explained in Experimental Procedures. Inhibitor binding causes global conformational changes in the protein, as indicated by significant differences between the two curves (Fig. 6A). Guinier plot analysis shows a reduction of the radius of gyration in the presence of the inhibitor from 25.0 to 20.7 (±0.2) Å, with a molecular mass estimate, derived from I(0)/c values, increasing from 28 to 29 kDa in the presence of 9a (Mw 1.4 kDa). Accordingly, in the distance distribution functions p(r) the maximal diameter D_max_ and radius of gyration R_g_ values undergo marked reductions, from 92 to 63 (±5) Å, and from 25.7 to 20.7 (±0.2) Å, upon inhibitor binding, respectively (Fig. 6B). All these results point toward a major conformational transition of XIAP-BIR2BIR3 from an extended (Fig. 6B inset, blue volume) to a more compact conformation (Fig. 6B inset, red volume) upon inhibitor binding, as already observed for a different divalent compound [16]. The p(r) profile of the apo protein is broadly spread with a first peak around 20 Å and a clear shoulder between 35 and 50 Å, corresponding predominantly to intra and interdomain distances, respectively (Fig. 6B, blue curve), while most distances are found in a narrow 10–45 Å range in the presence of the inhibitor with a much shorter D_max_ (Fig. 6B, red curve). This suggests that the two BIR2 and BIR3 structured domains are well separated in the absence of 9a, likely to be mobile around a flexible linker, while the divalent Smac-mimetic brings them into close proximity, resulting in the narrower distance distribution observed.

#### XIAP-BIR2BIR3 *ab initio* modeling

The shape of XIAP-BIR2BIR3 in the absence/presence of the inhibitor was investigated *ab initio* using the program Dammif [17]. We produced ten low-resolution models with/without 9a, all in excellent agreement with experimental data. Models superposition yielded values of *ca.* 0.85/0.75 of the normalized spatial discrepancy (NSD) for the protein in the absence/presence of the inhibitor, respectively, showing that all shapes in a series were very similar. The shape of XIAP-BIR2BIR3 in the absence of the divalent Smac-mimetic appears elongated and rather slim, large enough to accommodate the two BIR domains in non-contiguous positions together with the linker segment (Fig. 6B inset, blue volume). In contrast, the inhibitor-bound XIAP-BIR2BIR3 shows a broader, more compact shape that can accommodate the two domains in close proximity (Fig. 6B inset, red volume).

#### Apo XIAP-BIR2BIR3 modeling using crystal structures

Starting from a random mutual position of the two domains, the free XIAP-BIR2BIR3 construct was refined with respect to the SAXS data using the program Bunch [18], that modifies the relative position and orientation of the two domains while describing the missing parts (N- and C-terminal stretches together with the central linker) as chains of dummy residues (see Experimental Procedures section for details). Several models were obtained, nicely fitting the experimental data (χ = 0.9). After superimposition of the BIR2 domain from the various models, thus fixing the location of BIR2, the BIR3 domains and linkers appear to be widely distributed, with r.m.s.d. values over the BIR3 Cα atoms ranging between 11 and 56 Å (Fig. S1). Therefore, it appears that there is not a unique solution and that many different XIAP-BIR2BIR3 conformations can account for our data. All conformations exhibit domains at a moderate distance (13 Å to 19 Å between closest Cα atoms), suggesting that the molecule does not adopt a unique, well-defined structure but a manifold of conformations (Fig. S1). Indeed, the two domains are linked by a segment of 29 residues that is predicted to be extensively disordered and that likely provides substantial degrees of freedom for their mutual location. Accordingly, we submitted our data to analysis using the Ensemble Optimized Method (EOM) [19] that describes the sample as an ensemble of randomly created conformations (see Experimental Procedures). Panels C and D in Figure 6 show the distribution of values of R_g_ and D_max_ of the optimized ensembles compared to that of the starting pool. Both distributions show a major shift towards smaller values, indicating that XIAP-BIR2BIR3 adopts compact, less extended conformations more frequently than expected if the linker were oriented completely randomly. Such results suggest that XIAP-BIR2 and -BIR3 do not actually behave as independent domains, but are, most of the time, involved in some form of interaction even in the absence of 9a.

#### Modeling of complex XIAP-BIR2BIR3/9a using crystal structures

**Figure 7:**
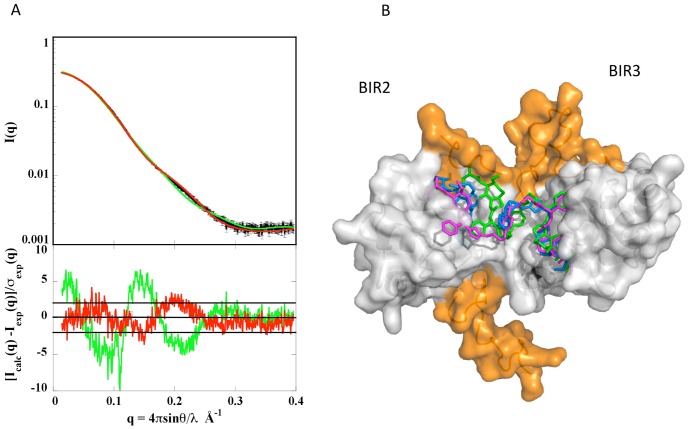
Scattering patterns and high resolution model of XIAP-BIR2BIR3 in presence of 9a. A) Experimental data with associated error bars is reported in black; green line: fit using Bunch and fixed domains with χ = 3.04; red line: fit using Coral and mobile domains with distant restraints between the two heads of 9a (χ = 1.30). B) Coral model: XIAP-BIR2BIR3 is represented in white/orange surface for BIR2BIR3/missing parts build by BUNCH, respectively. 9a is in blue stick and its conformation in XIAP- (shorter helical pitch) and cIAP1-BIR2BIR3 (longer helical pitch) crystal structures are in green/purple, respectively. The dimer that fit best SAXS data has somehow an intermediate structure with respect to the two observed crystal structures.

Starting from the crystal structure of the XIAP-BIR3 homo-dimer bound to 9a, we superimposed a BIR2 domain on one BIR3 domain thus producing an initial model of the interaction between the two domains in the presence of the ligand. We then used the program Bunch to model the missing parts according to the SAXS data, but no acceptable fit could be obtained. As a result, we undertook a new modeling stage using program Coral [20] in which domains BIR2 and BIR3 (each one bound to one half of 9a) were free to move under a 5 Å distance restraint between the two carbon atoms on each side of the broken methylbenzene bond in 9a. This made molecular sense in view of the numerous single bonds in the inhibitor offering as many possible rotations. Several models were obtained with a much-improved fit to the data (Fig. 7A with a χ-value of 1.3). However, the degrees of freedom of 9a make it likely that the XIAP-BIR2BIR3/9a complex exhibits a certain level of restricted mobility, so that the reported model (Fig. 7B) should be considered only as a representative of the ensemble of conformations explored by the molecule.

## Discussion

Compound 9a is a tail-tail homodimeric divalent Smac-mimetic that was rationally designed, together with nineteen other divalent compounds (tail-tail, head-head or head-tail, Fig. 1), to study how bifunctional inhibitors can bind and distinguish between XIAP- and cIAPs-BIR2BIR3 domains. Among these, 9a showed prominent binding activity to BIR3 domains of XIAP and cIAPs, and to XIAP-BIR2BIR3 (Table 1), low cytotoxicity in two different cell lines (MDA-MB-231 and HL60, Table 1), and the capability to induce activation of caspases and apoptosis (Fig. 2). Moreover, the divalent compound proved effective (alone) in *in vivo* treatments, after intraperitoneal daily administration, in two human IGROV-1 ovarian cancer models, showing reduction of subcutaneous tumor growth in *nude* mice, and increase of the median survival time of mice in ascites model [14].

Inspection of the crystal structures suggests that the higher affinity of 9a for cIAP1-BIR3, relative to XIAP-BIR3 (Table 1), is the result of: *i)* a larger IBM cleft, accommodating the ligand, due to Val/Leu292 and Asp/Glu314 residue substitutions in cIAP1/XIAP, respectively; *ii)* the presence of π-cation interactions stabilizing the ligand phenyl ring, due to Arg/Thr308 substitution (cIAP1/XIAP); and, *iii)* the increased negative charge located close to the ligand N-terminal end, due to Glu/Lys311 and Glu/Gln319 substitutions (cIAP1/XIAP) (Fig. 5A, B). Such features, promoting 9a affinity for cIAP1-BIR3, are partially compensated by the presence of Cys309 in cIAP1-BIR3, in place of Asp309, which in XIAP-BIR3 establishes an additional hydrogen bond with the ligand hydroxyl group located in the 4^th^-position of the azabicyclo[5.3.0]alkane scaffold.

Binding of the divalent compound to XIAP-BIR3 results in a crystal packing that differs from that observed in the crystal structures of the XIAP-BIR3 complexes with monovalent Smac-mimetic compounds known to date [11], [12], [16], [21], [22]. Notably, the crystal lattice packing is also different from that observed for XIAP-BIR3 in complex with the divalent compound-3 [16] (PDB: 3G76), whose crystal asymmetric unit hosts eight BIR3 molecules and eight compound-3 molecules, each of which has one inhibitory head bound to BIR3 and the other devoid of any contact to the protein. In contrast, 9a induces the formation of BIR3 dimers, in head-to-tail fashion, with a buried surface of about 652 Å^2^. Such dimers, not considering the 9a contributions, are stabilized by salt bridges and H-bonds mainly involving N-terminal residues Ser246, Asp247, Arg248, Ser253, and Arg258, and C-terminal residues His346, Ser347, Glu349, and Glu350 (analysis performed using the program ‘PISA’ [23]).

In the case of the cIAP1-BIR3/9a complex, the crystal packing matches that observed for cIAP1-BIR3 in the presence of the monovalent compound Smac037 [15]; thus, in this case, 9a (although conserving its overall right-handed helical conformation) apparently adapts its flexibility to a preferred crystallographic packing [15]. Such different behaviors observed for crystal packing are in keeping with the XIAP-BIR3 higher affinity for 9a complexed with one BIR3 domain, relative to that for the free inhibitor, as shown by gel filtration experiments. In fact, the higher affinity of XIAP-BIR3 for the bound ligand can be explained by the cooperation of two distinct contact interfaces, namely BIR3-BIR3 and BIR3-9a free head. Such hypothesis is supported by the existence of a larger interaction surface area for the XIAP-BIR3 dimer compared to cIAP1-BIR3, as assessed by the 'PISA' program [23] (association interface areas of 652/459 Å^2^, and ΔG^s^ of −6.9/−5.4 kcal/mol for XIAP/cIAP1-BIR3, respectively). Accordingly, the remarkable differences in affinity between XIAP- and cIAP1-BIR3 and the monomeric moiety of 9a (IC_50_ = 230.0 and 5.0 nM, respectively), are strongly reduced in the presence of the dimeric compound 9a (IC_50_ = 25.4 and 5.4 nM, respectively), confirming the ligand induced formation of an energetically favorable XIAP-BIR3 dimer (reducing the IC_50_ of the ternary complex).

Comparative SAXS analysis of XIAP-BIR2BIR3 shows that the construct in the presence of the inhibitor adopts a more compact global conformation, likely induced by 9a simultaneous binding to both BIR domains. However, ensemble analysis (EOM) of free XIAP-BIR2BIR3 shows that a majority of the molecules adopt a compact conformation, suggesting that the two domains are transiently interacting even in the absence of 9a. Such result is also supported by a molecular dynamics simulation of XIAP-BIR2BIR3 showing the conservation of an inter-domain interaction surface similar to that observed for XIAP-BIR3/BIR3/9a crystallographic dimer (data not shown).

A high resolution model of XIAP-BIR2BIR3/9a complex using the domain crystal structures that nicely fits SAXS data can be obtained by slightly relaxing the shape of the XIAP-BIR3/9a crystallographic dimer. In fact, a small separation of the two domains and the addition of the missing part of the structure (Coral model, Fig. 7B) lead to a much improved agreement with the SAXS data (χ decreases from 3.04 to 1.30). In this simulated model, 9a maintains a right handed helical conformation, but with a pitch that is intermediate relative to both cIAP1-BIR3 (longer) and XIAP-BIR3 (shorter) (Fig. 7B). The SAXS experimental evidence of the presence of a transient interaction between XIAP-BIR2BIR3, even when 9a is absent, indicates that the inhibitor may shift a preexisting equilibrium between open and closed conformations of the two domains toward the closed state. As a result, the overall affinity of XIAP-BIR2BIR3 for the compound would reflect both the mutual affinity of the two domains and the affinity of each domain for one 9a inhibitory head. On these bases, the design of an optimal divalent Smac-mimetic compound should take into account: *i)* the affinity of its heads for both BIR2 and BIR3 (homo or heterodimeric compounds); and, *ii)* the characteristics of the linker between the two heads, in particular considering its length, hydrophobicity and conformational freedom. Our structural results demonstrate that the 9a linker is well-suited to favor BIR2/BIR3 native mutual interactions in the ternary complex: both linker length (comparable with that of one active head) and conformational degrees of freedom allow 9a to adopt the observed right handed helical conformation with the two active heads mutually antiparallel. Moreover, the 9a linker hydrophobicity warrants an overall compact structure of the free ligand in solution (to minimize its hydrophobic surface), but with significant solvent exposure of the two active heads (antiparallel arrangement), as observed in molecular dynamics simulations of free 9a in solution (Fig. S2).

All results reported here emphasize the importance of structural dynamics in IAPs interactions with inhibitors and provide new hints for the development of divalent lead compounds able to bind preferentially XIAP, cIAP1 and cIAP2, thereby introducing specificity, albeit partial, in their action on different apoptotic pathways.

## Experimental Procedures

### Chemistry

The synthesis of lead divalent compound 9a was reported in details elsewhere [13]. Briefly, amidation of key intermediate **A** [24] with a phenyl-substituted propargylamine gave the alkynamide **B**, subsequently submitted to a click chemistry experimental protocol with a bifunctional azide reagent. The resulting bis-Boc protected tail-tail dimer **C** was finally deprotected in acidic conditions to provide pure 9a as a bis-trifluoroacetate in an overall ∼30% yield from A (Fig. S3).

### Cloning, Expression and Purification

The sequence coding for human XIAP-BIR2BIR3 (140–356) and cIAP1 and cIAP2-BIR3 (245–357) were cloned in pET21(b) (Novagen) with a C-terminal 6xHis-tag. The plasmids were used to transform *Escherichia coli* strain BL21(DE3). The recombinant proteins were purified using Ni-NTA (His-trap FFcrude, Ge-Healthcare), followed by gel filtration (Superdex 200, Ge-Healthcare). The elution buffers composition was 20 mM Tris pH 7.5, 200 mM NaCl, 10 mM DTT, and 20 mM Tris pH 8.0, 250 mM NaCl, 10 mM DTT for XIAP-BIR2BIR3 and cIAP1-BIR3, respectively. Finally, XIAP-BIR2BIR3 and cIAP1-BIR3 were concentrated to 10 mg ml^−1^ for crystallization tests using an Amicon Ultra centrifugal filter (10 kDa cut-off). XIAP-BIR3 domain was cloned, expressed and purified as already described [11].

### Fluorescence Polarization

Fluorescence polarization assays were performed according to Lu *et al.* [10], as also described in Cossu *et al*. ( [16]). Compound 9a was evaluated for its ability to displace the fluorescent probes (FITC-SMAC for BIR3 and SMAC-1F for BIR2BIR3) [11], [15]–[16] from the recombinant proteins. Fluorescence polarization was measured on an Ultra plate reader (Tecan), at excitation and emission wavelengths of 485 nm and 530 nm, respectively. All experiments were performed in black, flat-bottom 96-well microplates (Greiner bio-one).

### Analytical Gel Filtration Assays

In order to check the simultaneous interaction of compound 9a with the XIAP-BIR2 and BIR3 domains within the same protein molecule, analytical gel filtration was performed, using the construct XIAP-BIR2BIR3 (35 µM) alone or in the presence of the Smac-mimetic compound (1 mM). In solution, compound 9a ability to bind to two distinct BIR3 domains from cIAP1 or XIAP was also evaluated, using a protein concentration of 33 µM with an equal molar or an excess (1 mM) of 9a. Analytical gel filtration experiments were performed on a Superdex 200 column (GE Healthcare) attached to an AKTA Purifier-10 system in Tris-HCl, (pH 7.5, 20 mM), NaCl (200 mM) and DTT (10 mM). Low molecular weight standards from Amersham-Biosciences were used to calibrate the column.

### Cellular Cytotoxicity and in vitro-profiling

The MDA-MB-231, HL60 and PC-3 cell lines were obtained from Interlab Cell Line Collection (ICLC, Genova, Italy). All the cell lines were cultured at density of 1×105 cells per ml in RPMI 1640 medium supplemented with 10% fetal bovine serum (FBS) and at 37°C and 5% CO2 in fully humidified atmosphere. The effect of 9a on cell growth was evaluated by means of colorimetric assay for the quantification of cell proliferation and viability based on the cleavage of the WST-8 tetrazolium salt by mitochondrial dehydrogenases in viable cells (Promokine, Germany). The IC_50_, the concentration of compound capable of inhibiting the cell growth by 50%, was calculated using GraphPad Prism 4 software (see [14]).

### Caspase Activation and cIAP Degradation Cell-based Assays

To test the capability of the inhibitor to induce caspase activation and apoptosis, MDA-MB-231 cells were left untreated or treated with 100 nM of 9a, harvested, after 30 minutes and after 6 hours, and lysed. Proteins were revealed by Western blot using rabbit polyclonal antibodies specific for cleaved Parp, cleaved caspase-8, cleaved caspase-9 and cleaved caspase-3 (Cell Signaling), XIAP (BD Biosciences), cIAP1 and cIAP2 (R&DSystems), and mouse monoclonal anti-βactin (Sigma) as control.

### Crystallization and Crystallographic Data Reduction

Sitting drop crystallization experiments were prepared using an Oryx-8 crystallization robot (Douglas Instruments, East Garston, UK), from a 2∶1 mixture of the protein stock solution (XIAP-BIR3 or cIAP1-BIR3) with 1 mM 9a solution, and the precipitant solution, to a final drop volume of 0.3 µl for the initial screenings, and of 0.5 µl for the optimization trials. The screening solutions used for the experiments were those of Crystal Screens I & II and Index from Hampton Research (Aliso Viejo, CA, USA). After 1 day of vapour diffusion at 20°C, several irregular-aggregated crystals were obtained for both the proteins in complex with 9a. After optimization trials, some single and prismatic crystals of the complex cIAP1-BIR3/9a were observed in 6% PEG 3350, 0.1 M BisTRIS pH 5.2, and 0.2 M magnesium chloride. XIAP-BIR3 in complex with 9a crystallized in 30% PEG400, 0.2 M sodium citrate, and TRIS pH 7.5. The crystals obtained were soaked in a cryoprotectant solution containing 25% glycerol and flash-cooled in liquid nitrogen. The cIAP1-BIR3/9a crystals diffracted to a maximum resolution of 2.6 Å using synchrotron radiation on beam-line ID29, and the XIAP-BIR3/9a crystals to 3.3 Å on beam-line ID23-1, at the European Synchrotron Radiation Facility (ESRF-Grenoble, France). The diffraction data were processed with MOSFLM [25], and intensities were merged using SCALA [26].

### Structure Determination and Refinement

The cIAP1-BIR3/9a crystal belongs to the monoclinic C2 space group, with unit cell parameters a = 79.1, b = 81.3, c = 96.9 Å; β = 95.7°, with 4 molecules in the asymmetric unit (V_M_ = 2.8 Å^3^ Da^−1^, 55% solvent content [27]). The XIAP-BIR3/9a crystals belong to the orthorhombic P2_1_2_1_2_1_ space group, with unit cell parameters a = 77.7 Å, b = 108.4 Å, c = 225.3 Å, with 10 protein molecules in the asymmetric unit (V_M_ = 3.3 Å^3^ Da^−1^, 63% solvent content [27]). The crystal structures of cIAP1-BIR3 and XIAP-BIR3 in complex with 9a were solved by molecular replacement (‘molrep’ program [28]), using the structure of the BIR3 domain in the cIAP1-BIR3/Smac037 (PDB code 3MUP [15]) and in the XIAP-BIR3/Smac005 (PDB code 2CLX [11]) as search models, respectively. The 4 independent molecules (A–D) of cIAP1-BIR3/9a, and the 10 molecules (A–J) of XIAP-BIR3/9a, were subjected to rigid-body refinement, and subsequently refined using REFMAC5 [29] and BUSTER [30]. A random set comprising 5% of the data was omitted from refinement for R-free calculation. Manual rebuilding [31] and additional refinement [32] were subsequently performed. Inspection of difference Fourier maps at this stage showed strong residual density, located between the α3 helix and the main β-sheet, compatible with one of the two Smac-mimetic heads of the inhibitor for each molecule in the asymmetric unit, which were accordingly model-built. The density corresponding to the linker region between the two heads of 9a became evident after a few cycles of refinement, allowing prompt model-building of its molecular structure.

In the refined cIAP-BIR3/9a model, the first 12 N-terminal residues (241–253) and the last 2 C-terminal residues are disordered. In the XIAP-BIR3/9a model the N-terminal residues 248–253 were model built, but amino acids 241–247 and the last five C-terminal residues are disordered. Data collection and refinement statistics are summarized in Table 2. The stereochemical quality of the models was checked using the program Procheck [33] and is summarized in the Table 2. Atomic coordinates and structure factors for cIAP1-BIR3/9a and XIAP-BIR3/9a complexes have been deposited with the Protein Data Bank [34] with accession code 4EB9 and 4EC4 respectively.

### Small Angle X-Ray Scattering

X-ray scattering data were collected at the beamline SWING of Synchrotron SOLEIL (Gif-sur-Yvette, France). All measurements were performed at 10°C. The data were recorded using a CCD-based detector (AVIEX) with a sample-detector distance of 2.07 m, covering the range of momentum transfer 0.010<*q*<0.45 Å^−1^ (*q* = 4π sinθ/λ, where 2θ is the scattering angle and λ = 1.033 Å the wavelength of the X-rays). XIAP-BIR2BIR3 with and without 9a was studied in Tris-HCl buffer 20 mM pH 7.5, NaCl 200 mM and 10 mM DTT at protein concentrations between 1 and 7 mg/ml. Solutions were continuously circulated during data recording through the 1.5 mm diameter quartz capillary contained in an evacuated vessel using the automatic sample changer (Agilent) at a flow-rate ensuring an irradiation time of *ca* one second. Under these conditions, no radiation damage could be detected in preliminary tests. Measurements were also performed using the SE-HPLC instrument (Agilent) online with the SAXS measuring cell, a unique feature of the SWING beamline [35]. Briefly, 20 µl of a 7 mg ml^−1^ sample solution were loaded onto the column. Scattering of the elution buffer before void volume was recorded and used as buffer scattering to be further subtracted from all protein patterns. Two-second successive frames were recorded since the elution flow ensured that no protein was irradiated for more than 0.4 s.

Data were averaged after normalization to the intensity of the transmitted beam before buffer subtraction using the program package PRIMUS [36]. The forward scattering I(0) and the radius of gyration (R_g_) were evaluated using the Guinier approximation [37]. Frames recorded using the SE-HPLC over the monomer peak were analyzed individually before averaging the appropriate subset of frames that yield identical R_g_ and I(0)/c values. The corresponding concentration was determined using the UV absorbance detector from the HPLC system and the value of the protein absorbance at 280 nm ε^280^_1%_ = 20.85. The resulting curve was spliced with that of the most concentrated sample after scaling to protein concentration to yield a complete scattering pattern. The distance distribution function p(r) was determined using the indirect Fourier transform method as implemented in the program GNOM [38]. The molecular masses of the solutes were evaluated by comparison of the forward scattering with that of water recorded in the same capillary using the value of 0.001647 cm^−1^ for the theoretical scattering intensity of water at 10°C.

Low resolution shapes were determined using the program Dammif which describes the protein as a compact assembly of identical dummy atoms [17]. Ten models were calculated and superimposed using the Damaver suite of routines [39]. They were compared using the Normalized Spatial Discrepancy (NSD) [40], the smaller the NSD value the higher the similarity between models.

The program EOM (version 1.3) describes a flexible molecule in solution, using an ensemble of typically 50 conformations extracted from a very large (10,000) pool of conformations [19]. The conformer pool is constructed by connecting domains treated as rigid bodies by self-avoiding linkers, where the dihedral angles of the linkers in the C_α_–C_α_ space are selected randomly but biased to comply with the quasi-Ramachandran plot [41] and the model generated is free from steric clashes. A genetic algorithm progressively refines the composition of the ensemble so that the average scattering pattern of the molecular conformations within the ensemble fits the experimental data within error bars. The process was repeated 200 times and the distribution of the radius of gyration and the maximum diameter were calculated and compared with those derived from the entire starting pool. This comparison yields some global features of the conformational space explored by the molecule as probed by SAXS.

Models of both XIAP-BIR2BIR3 with and without the inhibitor were obtained using the program Bunch which moves domains as rigid bodies while describing the missing parts of the molecule (N and C extremities together with the intervening linker) as chains of dummy residues (DR) so as to fit the experimental scattering pattern [18]. Models of the conformation in solution of XIAP-BIR2BIR3 with 9a were obtained using the program Coral [20] starting from the high-resolution model of BIR2-BIR3 complexed with 9a. Here, Coral was used exactly as the program Bunch but allowed us to impose a 5 Å distance restraint between two atoms of the inhibitor on each side of a broken methylbenzene bond in the 9a central benzene. The core domains complexed with the corresponding inhibitor moiety were considered as rigid bodies while missing parts at both N- and C-ends (23 and 8 residue long respectively) and the central linker (29 residue long) were modeled as dummy residues (DRs) centered at Cα positions. The DR chains in resulting models were substituted with a polypeptide backbone and side-chains were added using the program SABBAC [42]. The connectivity of the split 9a molecule was restored using rotational degrees of freedom around single bonds. Finally the scattering pattern of the model was recalculated using Crysol [43].

## Supporting Information

Figure S1 **Five models of Apo XIAP-BIR2BIR3 obtained by the program Bunch.** All models are superimposed over BIR2 domain (dark grey) in the center of the figure. The spheres correspond to the dummy residues used by Bunch to represent the missing parts (N- and C-terminal ends and the central linker). BIR3 domains are seen in very different orientations and positions but relatively close to BIR2 (13 Å to 19 Å between closest Cα atoms).
(DOCX)

Figure S2 **Stable conformation of the free 9a in water.** Briefly, we run a molecular dynamics simulation using the program Gromacs (www.gromacs.org/) for 10 ns with 'gromacs' force field (1 fs time step) and periodic boundary conditions (with a box 33.1×41.4×37.5 Å^3^ filled with 1629 water molecules). Cluster conformational analysis shows the achievement of stable conformation (conserved till the end of the simulation) after ∼2.5 ns. Here is shown 9a structure after ∼4.3 ns as reference conformation in the equilibrium structural cluster; the free inhibitor adopt a compact left-handed helical assembly with antiparallel disposition of the two active heads; 9a is depicted in an orientation similar to that reported in Fig. 7 in the main text (drawn with Pymol).
(DOCX)

Figure S3 **Schematic procedures for the synthesis of 9a.**
(DOCX)
