# Supplementary figures and images for: Structural Insight into Inhibitor of Apoptosis Proteins Recognition by a Potent Divalent Smac-Mimetic
Source: PLoS One. 2012 Nov 15;7(11):e49527. doi: 10.1371/journal.pone.0049527 (PMC3499469; doi:10.1371/journal.pone.0049527)

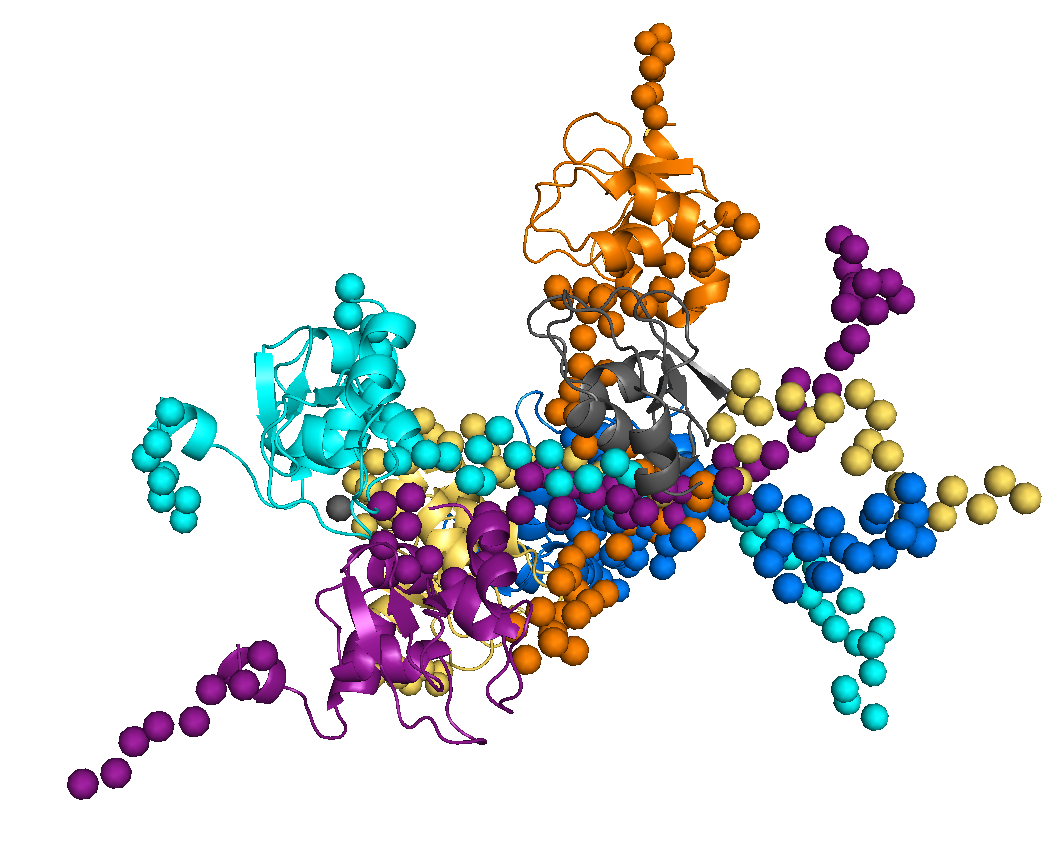


Figure S1

Supplement: Figure S1 — Five models of Apo XIAP-BIR2BIR3 obtained by the program Bunch. All models are superimposed over BIR2 domain (dark grey) in the center of the figure. The spheres correspond to the dummy residues used by Bunch to represent the missing parts (N- and C-terminal ends and the central linker). BIR3 domains are seen in very different orientations and positions but relatively close to BIR2 (13 Å to 19 Å between closest Cα atoms). (DOCX) [file pone.0049527.s001.docx]

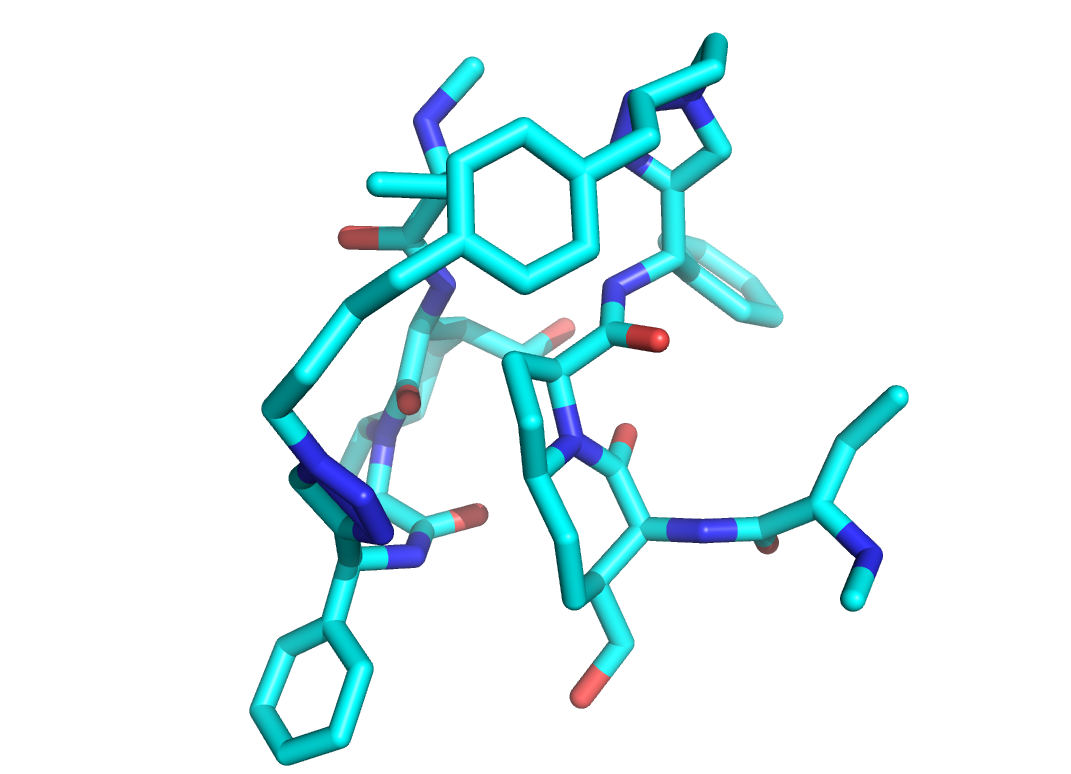


Figure S2

Supplement: Figure S2 — Stable conformation of the free 9a in water. Briefly, we run a molecular dynamics simulation using the program Gromacs (www.gromacs.org/) for 10 ns with 'gromacs' force field (1 fs time step) and periodic boundary conditions (with a box 33.1×41.4×37.5 Å3 filled with 1629 water molecules). Cluster conformational analysis shows the achievement of stable conformation (conserved till the end of the simulation) after ∼2.5 ns. Here is shown 9a structure after ∼4.3 ns as reference conformation in the equilibrium structural cluster; the free inhibitor adopt a compact left-handed helical assembly with antiparallel disposition of the two active heads; 9a is depicted in an orientation similar to that reported in Fig. 7 in the main text (drawn with Pymol). (DOCX) [file pone.0049527.s002.docx]
